# Perspectives and Misconceptions of an Online Adult Male Cohort Regarding Prostate Cancer Screening

**DOI:** 10.3390/curroncol31100475

**Published:** 2024-10-20

**Authors:** Tyler Sheetz, Tasha Posid, Aliza Khuhro, Alicia Scimeca, Sarah Beebe, Essa Gul, Shawn Dason

**Affiliations:** 1 Division of Urologic Oncology, Department of Urology, The Ohio State University Comprehensive Cancer Center, 2121 Kenny Road, Columbus, OH 43210, USAshawn.dason@osumc.edu (S.D.); 2 Department of Urology, University of California San Diego, 200 West Arbor Drive, San Diego, CA 92103, USA

**Keywords:** PSA, prostate cancer, PSA screening, USPSTF, CUA guidelines

## Abstract

Introduction: Congruent with most guideline publishers, the Canadian Urological Association (CUA) recommends shared decision-making (SDM) on PSA screening (PSAS) for prostate cancer (PCa) following a discussion of its benefits and harms. However, there are limited data on how the general male population feels about these topics. Methods: A survey was completed by 906 male-identifying participants (age > 18) recruited via Amazon Mechanical Turk (MTurk), which is a crowdsourcing platform providing minimal compensation. Participants answered questions regarding demographics (15), personal/family history (9), PCa/PSA knowledge (41), and opinions regarding PSAS (45). Results: The median age was 38.2 (SD = 12.0), with 22% reporting a family history of PCa and 20% reporting personally undergoing PSAS. Although most participants had heard of PCa (85%) and that they could be screened for it (81%), they generally did not feel knowledgeable about PCa or PSAS guidelines. Most want to talk to their clinician about PCa and PSAS (74%) and are supportive of SDM (48%) or patient-centered decision-making (25%). In general, participants thought PSAS was still worthwhile, even if it led to additional testing or side effects. Similarly, participants thought higher-risk patients should be screened earlier (*p* < 0.001). A number of misconceptions were evident in the responses. Conclusions: Men approaching the age of PSAS do not feel knowledgeable about PCa or PSAS and want their clinician to discuss these topics with them. The majority believe in PSAS and would like to undergo this screening following SDM. Clinicians also have a role in correcting common misconceptions about PCa.

## 1. Key Messages

Most adult men…

○ have low knowledge and possess misconceptions about prostate cancer and screening;
○ are interested in screening for prostate cancer, including PSA;
○ want to be included in shared decision-making for prostate cancer screening;
○ accept the negative trade-offs associated with screening, including unnecessary testing and treatment.

### 1.1. Social Media Blurb

We surveyed an online cohort of adult men regarding their knowledge, opinions, and attitudes about prostate cancer and PSA screening. This should help providers navigate the decision to screen in an era of multiple conflicting screening guidelines.

### 1.2. Introduction

Prostate cancer (PCa) is a significant public health concern, as it is the most commonly diagnosed non-cutaneous malignancy in men [1]. Most PCa is detected by screening men with serum prostate-specific antigen (PSA). The utilization of PSA screening (PSAS) for PCa in men started in the late 1980s and greatly increased the incidence of PCa [2]. Although epidemiologic data support a reduction in the PCa death rate by 50% since the 1980s, it is also evident that much of the PCa diagnosed with PSAS was not destined to be fatal. Meanwhile, most screen-detected cancers prompted radical treatment upon diagnosis, which is associated with significant sexual and urinary side effects [3,4].

PSAS has been increasingly scrutinized over the past decade for its role in the overtreatment of nonfatal cancers. Two large-scale studies of PSAS have been conducted—the US PLCO study and the European ERSPC study. At the time of their publication in 2009, the PLCO study did not show a PCa survival benefit with PSAS, while the ERSPC study suggested a very modest benefit. This prompted the United States Preventative Services Task Force (USPSTF) to recommend against PSAS in 2012, primarily on the results of the US-based PLCO trial [5]. This shift in USPSTF recommendations prompted changes in the practice pattern for PSAS, including a reverse PCa state migration with more advanced disease upon presentation [6,7].

More recent data have found that PLCO had a contaminated control arm, invalidating conclusions surrounding PSAS arising from this study [8]. In parallel, additional follow-up has demonstrated an even greater benefit of PSAS on prostate cancer mortality in ERSPC. In 2017, these updated perspectives on PSAS studies prompted the USPSTF to reinstate their recommendation for annual PSAS for average-risk men aged 55–69 with shared decision-making (SDM) between the patient and provider [9]. Other guideline bodies uniformly recommend SDM on whether to pursue PSAS. 

Introduced on a large scale in the 1990s, SDM is the idea that a patient and clinician will decide on a course of action after having a discussion surrounding the harms and benefits of a proposed intervention [10]. SDM has gained traction recently as global healthcare trends increasingly emphasize patient autonomy measures and patient-reported outcomes [11]. SDM is appropriate for most patients (and/or their families) in making important health decisions, especially in situations in which a decision must weigh distinct patient priorities (e.g., quality of life vs. cancer survival). While the adoption of SDM in the decision to conduct PSAS has been slow [12], it has increased with recent guideline endorsements, including support from the American Urological Association (AUA) [13,14,15], as well as the CUA [1].

Successful SDM is invariably impacted by a patient’s degree of disease-specific knowledge and health literacy [16]. Although public knowledge of PSAS was studied in the era before the 2017 guideline change [17,18,19,20,21,22,23,24], there are limited data regarding comprehension of PCa concepts in the era of the more supportive 2017 USPSTF recommendations. This is especially relevant as PCa information found online is rarely actionable, outdated, and contains misinformation [25,26,27]. Thus, we sought to survey men in the general population to investigate the knowledge, perspectives, and misconceptions regarding PCa and PSAS via an online survey-based platform. 

## 2. Materials and Methods

### 2.1. Survey

A 64-item survey instrument was developed internally by the Department of Urology. The survey queried participants on the following topics: Part 1: demographics (11 items), Part 2: personal/ family history (7 items), Part 3a: PCa general knowledge (13 items), Part 3b: personal PCa experience (14 items), Part 4a: PCa general opinion (21 items), Part 4b: PSAS opinion (2 items), and Part 4c: other opinion (8 items). See the Supplementary Materials for the full survey tool. The survey incorporated both rank order and multiple-choice formats. It underwent several rounds of review by the investigators, and a pilot version was administered to five undergraduate research assistants to gather initial feedback on the content, structure, organization, and clarity of the items.

### 2.2. Survey Distribution

The survey was distributed, and participants were recruited through Amazon Mechanical Turk (MTurk) (Amazon, Seattle, WA, USA), as in [28]. Amazon MTurk is an online crowdsourcing platform through which individuals complete tasks for modest compensation. Participants received a standard payment of USD 0.85 for completing the survey. Data collection and management were facilitated using Qualtrics, a web-based software platform employed for survey design, distribution, data storage, and obtaining informed consent. The survey was open for participation for 25 days. 

### 2.3. Study Sample

Survey data were collected from 906 participants, all of whom were men aged 18 years or older and located in the United States, as verified through IP address tracking. Participants provided informed consent for the use of their survey data for research prior to completing the survey. Although Amazon MTurk charges additional fees for specifying demographic criteria, no further restrictions (e.g., age) were set. However, these demographic metrics were tracked and analyzed as part of this study’s objectives. The demographics of the study sample, along with descriptive statistics, are presented in Table 1.

### 2.4. Comparative Cohort

To draw a comparison in the attitudes toward PSAS between middle-aged male patients and healthcare trainees and workers, we utilized a dataset from our previously published report investigating PSAS attitudes among healthcare trainees [29]. We split the two cohorts into the following three groups for comparative analysis: healthcare trainees, healthcare workers, and non-healthcare workers.

### 2.5. Statistical Analysis

All data analysis was performed using SPSS Statistics software (IBM SPSS Statistics for Windows, version 26.0. Armonk, NY, USA: IBM Corp). Descriptive data are presented as means (standard deviations) or proportions (percentages). For select questions, a single-sample *t*-test was performed comparing a single group’s mean value to “neutral” (or 3). Significant *p*-values (<0.05) indicate that respondents “strongly agree” (i.e., their answers were significantly above “neutral” or 3).

## 3. Results

### 3.1. Demographics

Demographic data are listed in Table 1. The mean age in the survey cohort was 38.2, with a standard deviation of 12, indicating that between 30 and 50% of men would fall within the PSA screening age range (depending on the guideline used). Most of the cohort not in the PSAS age range would have been confronted with this topic in the next decade. Race was self-identified as White in 68.3%, Asian in 17%, non-white Hispanic or Latino in 17%, and Black in 10%. The cohort was educated, with 80% possessing a postsecondary degree. Most participants’ highest education level was a bachelor’s degree (54%), followed by a secondary school degree (19%), and then a graduate or professional degree (18%). About one-third of the cohort indicated working in healthcare, and only 27% were active smokers, suggesting that overall health literacy and awareness may be above average. Furthermore, participants classified their overall health at a mean value of 7.4 (1.7) on a 10-point scale, and 73% reported going to the doctor for a physical at least yearly. 

As for prostate-specific descriptors, 22% reported a family history of PCa, and 20% have previously undergone PSAS. A total of 7% of the cohort had been diagnosed with PCa in the past.

### 3.2. Prostate Cancer Knowledge

As seen in Table 2a, men were asked about their perceived personal knowledge of prostate cancer and PSAS. Although most men had heard of PCa (85%) and that they could be screened for it (81%), less than 30% felt knowledgeable about PCa or PSAS guidelines. The average response regarding self-reported PCa knowledge was 2.7/5 (less than a “neutral” response of 3 *p* < 0.001). 

For objective measures listed in Table 2b, multiple-choice assessment questions were embedded within the survey. The cohort performed poorly overall on an assessment that focused on basic knowledge of PCa, with an average percent correct of 44% across the entire assessment. For example, most participants (74%) answered correctly that PCa is treatable; however, only 22% answered correctly about the appropriateness of treating PCa. Overall, a number of misconceptions were evident in the responses (Table 3).

### 3.3. Prostate Cancer Screening Attitudes

**Figure 1:**
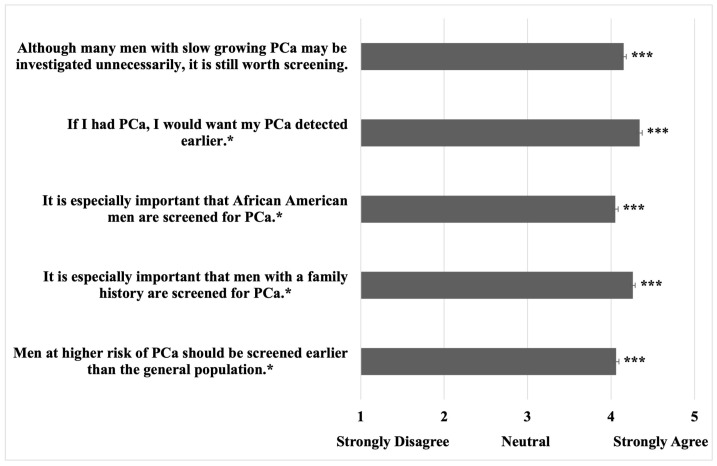
Perspectives of middle-aged men regarding PCa screening; * survey text contained additional background information in the question stem relevant to the question; *** *p* < 0.001 vs. “neutral” response.

Survey responses pertaining to PCa attitudes and opinions are displayed in Table 4. The majority of men want to talk to their clinician about PCa (71%) and PSAS (74%), and 49% are supportive of SDM for PSAS. Furthermore, 62% of patients would be upset if their doctor did not discuss PSAS with them. Additionally, 41% of men thought PSAS was worthwhile in all patients for detecting PCa (even if it led to additional testing or side effects), and an additional 34% thought PSAS was worthwhile for high-risk patients. Likewise, participants thought that higher-risk men should be screened earlier (mean 1.75/5, SD = 0.88, *p* < 0.001 vs. neutral response), including Black men and those with a positive family history (Figure 1).

**Figure 2:**
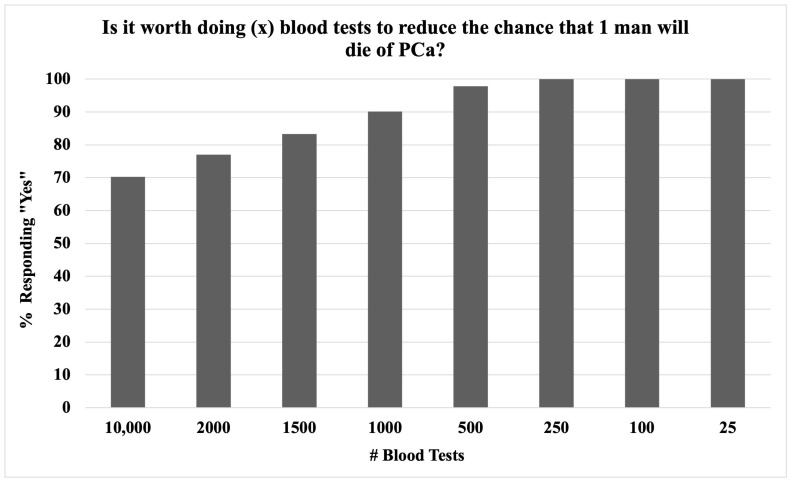
Exploring the threshold for the number needed to screen to reduce the chance that one man would die from PCa in middle-aged men; 100% of participants agreed that it was worth screening 250 patients to reduce the risk of one PCa death; PCa = prostate cancer, PSA = prostate-specific antigen, PSAS = PSA screening.

In an attempt to delineate a generally agreed-upon threshold of risk/benefit of PSAS among our cohort, we asked participants how many men would be acceptable to screen with a blood test to reduce the risk that one man would die of PCa. As seen in Figure 2, the majority of participants (70%) thought it was worth screening 10,000 men with a blood test to reduce the risk that 1 man would die. The threshold number of blood tests all participants agreed it was worth performing to reduce the risk that one man would die was 250.

**Figure 3:**
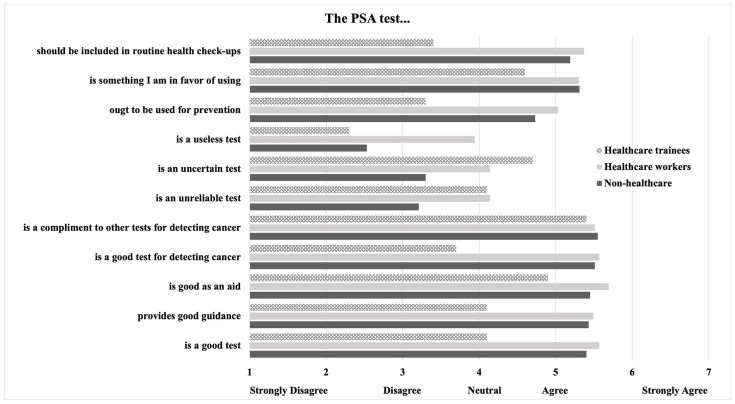
Comparison of middle-aged male mTURK cohort to a healthcare trainees cohort [29] regarding PSAS perspectives on a Likert scale of 1 (strongly disagree) to 7 (strongly agree); PSA = prostate-specific antigen, PSAS = PSA screening.

As mentioned, we then utilized a dataset from our previously published report on PSAS attitudes among healthcare trainees for a comparative analysis [29]. As seen in Figure 3, healthcare trainees generally regarded the PSA test less favorably than current healthcare workers and non-healthcare workers.

## 4. Discussion

Here we present a large survey study assessing the knowledge, opinions, and misconceptions of a population of predominantly middle-aged laymen. The majority of men in the cohort were either below or had just entered the recommended age range for PSAS, and 20% had undergone screening. Thus, this cohort represents a population of men who should be beginning to think about preventive care. As noted in Table 1, the population was well-educated, with 80% possessing a postsecondary degree, highlighting that misconceptions about PCa and PSAS are prevalent, even in those with advanced degrees.

Although PCa knowledge was low, with an average score on our summative basic PCa knowledge assessment of 44%, this well-educated middle-aged male cohort felt favorably about PSAS in general. This notion was supported in the results, with answers to several questions indicating that most men want to discuss PSAS with their doctor (and many would even be upset if not discussed). Interestingly, most men accepted the negative trade-offs associated with screening, including unnecessary testing and treatment, with 70% of participants willing to screen 10,000 men to reduce the risk of death of 1 man from PCa, reflecting an aggressive screening strategy by current standards. These results emphasize the importance of education in making informed decisions about screening. We suspect this high acceptance of screening in spite of a limited screening benefit was in part due to some of the misconceptions about PCa listed in Table 3. For example, 71% of participants were not aware that PCa may not require treatment, and 27% thought that PCa is always or usually fatal (Table 3). Overall, the findings of this paper support the recently published AUA/SUO guidelines that advocate for earlier PSAS with SDM, stratifying patients by risk category [15].

When attitudes regarding PSAS were compared between the current cohort and a prior cohort of medical students and residents at a large public university surveyed by the authors [29], we found that the general population feels much more favorably about PSAS compared to the medical student and resident cohort (Figure 3). The prior survey was administered in 2019, only 2 years after the USPSTF revised its controversial recommendations around PSAS, which may explain some degree of the negative attitude displayed by trainees. In the era of the prior survey, medical trainees were undoubtedly exposed to a significant negative perspective on PSAS, resulting from the 2012 USPSTF recommendations against PSAS. To reflect the viewpoints of their patients, our current survey findings emphasize that healthcare workers trained during the tumultuous PSAS guideline environment of the 2010s may need further education regarding the utility of PSAS in men aged 55–70 and shared decision-making.

The possible limitations of this study include the fact that the majority of the individuals in the dataset are Caucasian and have a bachelor’s degree, indicating a potential bias toward this demographic when applied in different settings [30]. The individuals in the dataset have a relatively low smoking rate (27%) (Table 1), which may suggest that this population has a higher level of health awareness relative to the general population and could indicate that the degree of disparity in PSAS education elucidated in this study could be underrepresented. The low percentage of individuals who have received PSA screening, DRE screening, and other PCa diagnostic (Figure 1) testing may suggest that the population of interest was not optimally captured in this survey. This is likely due to the younger population that was captured (likely due to the digital nature of the survey) at a median age of 38 years (>80% between the ages of 20 and 50 years). Nevertheless, this may represent a population in which men should begin to be educated about screening for PCa and colorectal cancer, which should begin to be discussed at the age of 40–45 per current AUA/SUO guidelines [15]. Lastly, during survey development, the survey was vetted by undergraduate research assistants, which could have impacted the ability of older men to complete it, although it was edited only for content, structure, organization, and clarity of the items.

Potential future directions include an analysis comparing/contrasting findings in men greater and less than the age typically recommended for PSAS. Given that women make the majority of family medical decisions, surveying women who have middle-aged men in the family would be interesting and useful. Additionally, further investigating attitudes, opinions, and knowledge of minority racial/ethnic groups toward PSAS may be interesting and help develop targeted education programs, especially for the higher-risk Black male subset. Finally, using the survey tool to measure gains in knowledge after education may potentially identify opportunities and areas of focus to improve the SDM process.

## 5. Conclusions

An online cohort of young adult well-educated men have low levels of baseline knowledge regarding prostate cancer and PSA screening, and there are a number of misconceptions evident in the high prevalence of the facts. These men generally feel favorably regarding PSAS and are willing to accept risks, overdiagnosis, and overtreatment for a chance at catching prostate cancer earlier and potentially reducing prostate cancer mortality. This is in contrast to medical trainees surveyed in 2019, who were more skeptical of the PSA test. Nevertheless, most adult men approaching the age of PSA screening want to at least discuss screening with their provider, and some would even feel upset if it was not discussed.

## Figures and Tables

**Table 1:** Demographic variables displayed for the study cohort (n = 906); DRE = digital rectal exam, PCa = prostate cancer, PSA = prostate-specific antigen, PSAS = PSA screening.

Demographics (n = 906)	
Variable	Mean (SD) or n (%)
Age (years) 20–29 30–39 40–49 50–59 60–69 70–79	38.2 (12.0) 226 (24.9%) 333 (36.8%) 200 (22.1%) 77 (8.5%) 52 (5.7%) 14 (1.5%)
Married Status	498 (55.0%)
Median Yearly Household Income Category	USD 50,000–74,999
Race/Ethnicity	
Caucasian	619 (68.3%)
Asian	151 (16.8%)
Hispanic or Latino	150 (16.6%)
Black	88 (9.8%)
Other	21 (2.3%)
Highest Degree Level	
Less than a secondary school degree	4 (0.4%)
Secondary school degree (or equivalent)	174 (19.2%)
Associate degree	58 (6.4%)
Bachelor degree	491 (54.2%)
Professional degree (e.g., J.D. or M.D.)	47 (5.2%)
Graduate degree (e.g., Ph.D.)	115 (12.7%)
General Health Descriptors	
Healthcare worker	266 (29.4%)
Current health self-rating (1–10 Likert scale)	7.4 (1.74)
Current smoker	247 (27.3%)
Prostate Health Descriptors	
Ever received PSAS?	181 (20.0%)
Ever received DRE screening?	227 (25.1%)
Ever been told your PSA was elevated?	152 (16.8%)
Ever received PCa diagnostic testing?	149 (16.4%)
Ever been diagnosed with PCa?	64 (7.1%)
Ever had a family member diagnosed with PCa?	201 (22.2%)
Ever had a family member die from PCa?	113 (12.4%)

**Table 2:** Prostate cancer knowledge assessed (**a**) subjectively via self-evaluation questions and (**b**) objectively via summative questions built into the survey; PCa = prostate cancer, PSA = prostate-specific antigen, PSAS = PSA screening.

2a. Self-Reported Prostate Cancer Knowledge.	
Survey Question	Affirmative Answer, n (%)
Do you know…	
…what a prostate is?	768 (84.8%)
…that men can get PCa?	773 (85.3%)
…that men can get screened for PCa?	733 (80.9%)
…how men get screened for prostate cancer?	538 (59.4%)
Do you possess little or no knowledge about…	
…PCa?	361 (39.8%)
…PSAS guidelines?	574 (63.3%)
**2b. Prostate Cancer and PSA Multiple-Choice Knowledge Assessment.**	
**Assessment Question**	**Correct Answer, n (%)**
Is PCa treatable?	672 (74.2%)
What are the side effects of PCa treatment?	421 (46.5%)
How common is PCa?	315 (34.8%)
Which age group is most likely to develop PCa?	234 (25.8%)
Does all PCa require treatment?	196 (21.6%)
Is PCa fatal?	549 (60.9%)
Average correct	44.00%

**Table 3:** Table of most prevalent incorrect answers from the summative questions organized by order of prevalence (correct answers not displayed); PCa = prostate cancer.

Misconceptions Regarding Prostate Cancer (PCa) Among Middle-Aged Men		
Survey Question	Incorrect Answers	n (%)
Does all PCa require treatment?	Yes	644 (71%)
What age group is most likely to get PCa?	41–60	416 (46%)
	21–40	154 (17%)
	0–20	10 (1%)
How common is PCa?	Top 5 male cancer	373 (41%)
	Rare	128 (14%)
	Top 20 male cancer	81 (9%)
Chance a man will develop PCa in his life?	Pretty low chance	209 (30%)
	Very low chance	113 (16%)
Is PCa fatal?	Usually	182 (20%)
	Always	60 (7%)
	Never	66 (7%)
Can men get screened for PCa?	No	164 (18%)
Can men get PCa?	No	122 (14%)
Is PCa treatable?	Always	121 (13%)
	No	77 (9%)

**Table 4:** Prostate cancer and PSA screening perspectives and preferences; * survey text contained additional background Information in the question stem relevant to the question; Dr. = doctor, PCa = prostate cancer, PSA = prostate-specific antigen, PSAS = PSA screening.

Prostate Cancer and PSA Screening: Perspectives and Preferences		
Survey Question		Affirmative Answer, n(%)
Has your doctor talked to you about PCa?		
	No	526 (58.1%)
	Once	239 (26.4%)
	More than once	115 (12.7%)
Would you like your doctor to talk to you about PCa?		
	No	262 (28.9%)
	Regularly	251 (27.7%)
	One time	244 (26.9%)
	Already do/did	118 (13.0%)
Would you feel upset if your Dr. did not talk to you about PCa?		276 (30.5%)
If screening existed, * would you…		
	want your Dr. to discuss it with you?	668 (73.7%)
	be upset if your Dr. did not discuss it with you?	561 (61.9%)
If I was diagnosed with PCa, this would cause me distress.		700 (77.3%)
Who should make the decision about whether or not a patient should have a PSA test?		
	Provider and patient together	442 (48.8%)
	Patient	230 (25.4%)
	I do not know	141 (15.6%)
	Provider	93 (10.3%)
Do you still think it is worth screening for PCa if…		
…PSAS could lead to side effects and even unnecessary treatment? *		
	Yes – always	372 (41.1%)
	Yes – for high-risk patients	305 (33.7%)
	It depends on the number helped vs. harmed	139 (15.3%)
	No	46 (5.1%)
…PCa treatment could cause erectile dysfunction?		
	Yes	669 (73.9%)
	No	172 (19.0%)
…PCa treatment could cause urinary problems (e.g., leakage)?		
	Yes	676 (74.6%)
	No	166 (18.3%)

## Data Availability

The original data presented in this study are openly available in the FigShare data repository at DOI [10.6084/m9.figshare.27122451].

## Supplementary Materials

The following supporting information can be downloaded at https://www.mdpi.com/article/10.3390/curroncol31100475/s1: File S1. Study Survey.
