# Supplementary material for: Perspectives and Misconceptions of an Online Adult Male Cohort Regarding Prostate Cancer Screening
Source: Curr Oncol. 2024 Oct 20;31(10):6395–405. doi: 10.3390/curroncol31100475 (PMC11506613; doi:10.3390/curroncol31100475)
Supplement: Supplementary file 1 [file curroncol-31-00475-s001.zip › curroncol-3205621-supplementary.pdf]

Screening Question

Are you a citizen of the United States of America? y/n

Part 1. Please fil out the following demographic information:

1. Age (years): _____
2. Gender:
   a. Male
   b. Female
   c. Other
3. Race:

   a. White

   b. Black or African American

   c. Asian

   d. American Indian/Alaska Native

   e. Native Hawaiian or Other Pacific Islander

   d. Two or more races

4. Ethnicity
   a. Hispanic or Latino
   b. Not Hispanic or Latino
5. Marital Status:
   a. Single
   b. In a relationship but not married
   c. Married
   d. Not married but living with a partner
   e. Divorced/separated
   f. Widowed
6. Highest level of education:
   a. Less than high school degree
   b. High school graduate or degree (for example, GED)
   c. Some college, no degree
   d. Associate degree
   e. Bachelor degree
   f. Non-healthcare professional degree (eg. JD)
   g. Healthcare-based professional degree (eg. MD, PharmD)
   h. Graduate degree (non-professional masters or PhD)
7. What is your yearly household income?
   a. Less than $20,000
   b. $20,000 - $34,999
   c. $35,000 - $49,999
   d. $50,000 - $74,999

    e. $75,000 - $99,999
    f. $100,000 - $149,999
    g. $150,000 - $199,999
    h. $200,000 or more
8. Do you currently work in the medical or healthcare field?
    a. Yes (if yes: Occupation: __________)
    b. No
9. Does anyone in your immediate family work in the medical or healthcare field?
    a. Yes (if yes: Relationship to you: __________; Occupation: __________)
    b. No
10. Do you have health insurance?
    a. Yes
    b. No
11. Who is your health insurer?
    a. Medicare
    b. Medicaid
    c. Private health insurance through employer or family member's employer
    d. Private health insurance obtained independent of employer
    e. Other government insurance
    f. Multiple
    g. Uninsured

Part 2. Personal/Family History

1. On a scale of 1-10 (1=bad, 10=very good), how would you classify your current overall health? __________
2. How often do you go to the doctor for regular physicals?
    a. Less frequently than every 6 months
    b. Every 6 months
    c. Once per year
    d. Every few years
    e. Never
3. Have you had your blood pressure checked within the past year?
    a. Yes
    b. No
4. Have you had your cholesterol checked within the past year?
    a. Yes
    b. No
5. Have you been checked for diabetes within the past year?
    a. Yes
    b. No
6. Have you had a colonoscopy or other test to screen for colon cancer?
    a. Yes
    b. No

7. Do you smoke?
    a. Yes
    b. No

If yes: has your physician talked to you about quitting smoking in the past 2 years?

a. Yes
b. no

Part 3: Prostate cancer general

Attention check: ***The color test is simple, when asked for your favorite color you must enter the word vermilion.***

Based on the instructions above, what is your favorite color?

1. Do you know what the prostate is?
    a. Yes
    b. No
2. Have you heard that men can get cancer of the prostate?
    a. Yes
    b. No
3. Have you heard that men can get screened for cancer of the prostate?
    a. Yes
    b. No
4. How common is prostate cancer?
    a. Most common male cancer
    b. Top 5 male cancers
    c. Top 20 male cancers
    d. Rare
5. Do you know how men get screened for prostate cancer?
    a. Yes
    b. No

If yes: please specify

6. Which age group is most likely to develop prostate cancer?
    a. 0-20
    b. 21-40
    c. 41-60
    d. 60+
    e. Don't know
7. Can you think of any symptoms that might suggest someone has prostate cancer?
8. Is prostate cancer treatable?
    a. Always
    b. Usually
    c. No
9. Is prostate cancer usually fatal?

    a. Always
    b. Usually
    c. Sometimes
    d. Never
10. Does all prostate cancer require treatment?
    a. Yes
    b. No
11. What are the side effects of prostate cancer treatment?
    a. Urinary problems
    b. Sexual problems
    c. Bowel problems
    d. None of the above
    e. All of the above

12. How knowledgeable do you feel about prostate cancer?
    a. Not at all knowledgeable
    b. Somewhat unknowledgeable
    c. As knowledgeable as others
    d. Somewhat knowledgeable
    e. Very knowledgeable
13. What is your current level of knowledge about prostate cancer screening guidelines?
    a. No knowledge at all
    b. A little knowledge
    c. A moderate amount of knowledge
    d. A great deal of knowledge

Personal questions

14. Have you personally received a PSA (prostate-specific antigen) blood test to screen for Prostate Cancer?
    a. Yes
    b. No

If yes: how often do you receive a PSA (once, yearly, every other year, less frequently)
If yes: at what age did you receive your first PSA?

15. Have you personally received a rectal exam to screen for prostate cancer?
    a. Yes
    b. No

16. Have you ever been told that your PSA is elevated?
    a. Yes
    b. No
17. Have you ever had any further testing to diagnose prostate cancer (e.g. MRI, biopsy)
    a. Yes

b. No

18. Have you ever been diagnosed with Prostate Cancer?
    a. Yes
    b. No

If yes: what age were you diagnosed at?
If yes: What treatment did you have (surgery, radiation, medications, monitoring)
If yes: are you currently cancer free?

19. Has a family member been diagnosed with Prostate Cancer?
    a. Yes
    b. No

If yes: Indicate relationship and age at diagnosis; allow multiple fields

If yes: did a family member die of prostate cancer?

20. If a close family member told you that their physician recommended that they get a PSA screening, would you agree with this:
    a. Yes, always
    b. Yes, but only if they are at high risk
    c. No
    d. Prefer not to answer/Don't know

    Why or why not: ____________

21. Has a family member received a PSA screening for Prostate Cancer that you are aware of?
    a. Yes
    b. No
    c. Prefer not to answer
22. If you have heard about the controversy and/or changes in PSA screening recommendations over the past decade or so, where did hear about this: (Choose all that apply)
    a. Environment
    b. Health care personnel
    c. In the classroom or in a text book
    d. Internet
    e. Tv/Media
    f. Other: ___________
23. Has your doctor talked to you about prostate cancer?
    a. Yes, more than once
    b. Yes, once
    c. No
24. Would you like your doctor to talk to you about prostate cancer?
    a. As indicated above, my doctor and I have already discussed this
    b. Yes, I would like to talk about this regularly (e.g. once per year)
    c. Yes, I would like to talk about this one time
    d. No
25. Would you feel upset if your doctor did NOT talk to you about prostate cancer?

   a. Yes
   b. No
26. If a screening test existed that may reduce the chance that you may die from prostate cancer, would you want your doctor to discuss this topic with you?
    a. Yes
    b. No
27. If a screening test existed that may reduce the chance that you may die from prostate cancer, would you be upset if your doctor did not discuss this topic with you?
    a. Yes
    b. No

Part 4. General Opinion

1. What do you think is the chance that you will develop prostate cancer in your lifetime?
   a. Very low chance
   b. Pretty low chance
   c. Neutral
   d. Pretty high chance
   e. Very high chance
2. Men with a family history of prostate cancer are known to have a higher risk of developing prostate cancer and a higher death rate from prostate cancer. Given these facts, it is especially important that men with a family history are screened for prostate cancer.
   a. Strongly agree
   b. Mildly agree
   c. Not sure
   d. Mildly disagree
   e. Strongly disagree
3. Black men are known to have a higher risk of developing prostate cancer, a higher death rate from prostate cancer, and many barriers in access to prostate cancer care. Given these facts, it is especially important that black men are screened for prostate cancer.
   a. Strongly agree
   b. Mildly agree
   c. Not sure
   d. Mildly disagree
   e. Strongly disagree
4. Men at higher risk of prostate cancer (e.g. those with a family history or black men) should be screened earlier than the general population
   a. Strongly agree
   b. Mildly agree
   c. Not sure
   d. Mildly disagree
   e. Strongly disagree

5. Although treatment is not perfect, early detection of prostate cancer allows for the avoidance of progression to more advanced disease and death. If I had prostate cancer, I would want my prostate cancer detected early
    a. Strongly agree
    b. Mildly agree
    c. Not sure
    d. Mildly disagree
    e. Strongly disagree
6. Although many men with slow growing cancer may be investigated unnecessarily, it is worth doing a screening test to pick up the few men with more aggressive disease:
    a. Strongly agree
    b. Mildly agree
    c. Not sure
    d. Mildly disagree
    e. Strongly disagree
7. Who should make the decision about whether or not a patient should have a PSA test?
    a. Provider
    b. Patient
    c. Provider and patient together
    d. I don't know
8. Prostate cancer can be screened for with a simple blood test (PSA test) but you have to screen many people to reduce the chance that someone will die from prostate cancer. Do you think it is worth doing blood tests for 10,000 people to reduce the chance that 1 will die?
    a. Yes
    b. No

If no→ Do you think it is worth doing blood tests for 2000 people to reduce the chance that 1 will die?

If no→ Do you think it is worth doing blood tests for 1500 people to reduce the chance that 1 will die?

If no→ Do you think it is worth doing blood tests for 1000 people to reduce the chance that 1 will die?

If no → Do you think it is worth doing blood tests for 500 people to reduce the chance that 1 will die?

If no → Do you think it is worth doing blood tests for 250 people to reduce the chance that 1 will die?

If no → Do you think it is worth doing blood tests for 100 people to reduce the chance that 1 will die?

If no → Do you think it is worth doing blood tests for 25 people to reduce the chance that 1 will die?

9. Many men diagnosed with prostate cancer will receive treatment that will cause urinary and sexual problems. Many of these men would never have died of their prostate cancer as a lot of prostate cancers are not aggressive. Do you think it is still worth screening for prostate cancer?

a. Yes – always
b. Yes – for high risk patients
c. It depends on how many men will have urinary and sexual problems vs. how many less men will die of prostate cancer
d. No

10. If I was diagnosed with prostate cancer, this would cause me distress
a. Yes
b. No
11. If I had a positive screening test but was not ultimately diagnosed with cancer on further testing, I would have been distressed until I got the final results
a. Yes
b. No
12. If I had some abnormal testing and my doctors were not sure if I had cancer or not, I would have ongoing distress
a. Yes
b. No
13. If I was diagnosed with cancer that was not aggressive and could be safely watched or treated, this would cause me distress
a. Yes
b. No
14. If I was diagnosed with a cancer that required treatment which would cure me but would impact my sexual and urinary function, this would cause me distress
a. Yes
b. No
15. If I was diagnosed with a cancer that could be controlled for many years but was not likely to be cured and would impact my sexual and urinary function, this would cause me distress
a. Yes
b. No
16. Do you think it is worth screening for prostate cancer if those diagnosed with prostate cancer have a chance of developing erectile dysfunction from treatment?
    a. Yes
    b. no
17. Do you think it is worth screening for prostate cancer if those diagnosed with prostate cancer have a chance of developing urinary problems (e.g. leakage) from treatment?
    a. Yes
    b. No
18. If you can prevent 1 person from dying from prostate cancer, how many people developing permanent erectile dysfunction is an acceptable tradeoff?
19. If you can prevent 1 person from dying from prostate cancer, how many people developing permanent urinary leakage is an acceptable tradeoff?
20. Consider how well you agree with the following statements about PSA testing:

1=Disagree, 2=Somewhat disagree, 3=Slightly disagree, 4=neutral, 5=slightly agree, 6=somewhat agree, 7=Agree

The PSA is:
A good test: _____
Provides good guidance: ______
Is good as an aid: _____

Is a good test for detecting cancer: _______
Is a compliment to other tests for detecting cancer: ______
Is an unreliable test: ______
Is an uncertain test: _____
Is a useless test: _____
Ought to be used for prevention: _____
Is something that I am in favor of using: _____
Should be included in routine health check-ups: _____

21. How aware are you of the controversy/recent recommendation changes about whether prostate cancer should be screened routinely (using PSA)?
    a. I am not aware of any controversy
    b. I am aware that there has been a controversy, but did not know recommendations had changed regarding PSA screening
    c. I am aware that there has been a controversy and did know that recommendations had changed regarding PSA screening

Early screening

1. Would you consider being screened for prostate cancer?
a. Yes
b. No

If no: please specify why

2. We know that screening men earlier (e.g. in their 40s) instead of the usual practice of screening men in their 50s can be beneficial in deciding how often to screen men and can also predict who is most likely to develop prostate cancer. When would you want to be screened?
A. 40s
B. 50s
3. We have more evidence that screening men starting in their 50s rather than in their 40s reduces death from prostate cancer. With this being said, when would you want to be screened?
a. 40s
b. 50s
4. If you had a higher risk of prostate cancer due to race or family history and your doctor didn't have the evidence of when it is best to start screening you, when would you want to be screened?
a. In my 40s
b. In my 50s
5. Earlier screening may lead to more invasive tests and it is unproven that this will reduce prostate cancer deaths. Given this, when would you want to be screened?
a. In my 40s
b. In my 50s
6. Earlier screening may lead to an earlier cancer diagnosis and more years living with sexual and urinary problems when this is treated. It is unproven that this earlier diagnosis would reduce prostate cancer death rate. Given this, when would you want to be screened?

a. In my 40s
b. In my 50s

Other questions

1. There isn't great evidence as to whether men should be screened yearly, every other year, or every few years. Do you have a preference on how often you would want to be screened?
a. Yearly
b. Every other year
c. Every few years
2. There isn't great evidence that doing a rectal exam improves prostate cancer screening. Do you have a preference on whether you want to be screened with a rectal (prostate) exam?
a. Yes
b. Only if my doctor feels it is appropriate
c. No
3. Screening generally stops for men in their 70s because it is thought that causes of death other than prostate cancer are more likely once you get into this age range. That being said, many men may still live for 10-20 years and prostate cancer could still be an important issue for them. Given this, would you want to be screened for prostate cancer after 70?
A. Yes
B. Only if my doctor feels it is appropriate
C. No
4. If you were suspected to have a risk of prostate cancer, the next step could be a MRI or a biopsy. A MRI would allow 1 in 3 men to avoid a biopsy and involves some cost as well as sitting in a claustrophobic machine for 45 minutes. A biopsy involves a rectal probe being placed and 10-14 needle cores of prostate tissue are extracted after local anesthetic is administered. A biopsy is still required if a MRI is positive. Would you rather have a MRI first or go straight to biopsy?
A. MRI
B. Biopsy
5. If a MRI is "negative" there is still a small chance that a cancer can be present that is invisible to the MRI. The only way to diagnose this cancer is to still do a biopsy even when the mri is negative. What is the maximum risk of cancer that you could accept without going for a biopsy?
a. 1%
b. 5%
c. 10%
d. 20%
e. 30%
f. 40%
6. A MRI would allow 1 in 3 patients to avoid a biopsy which may be uncomfortable and have associated risks of infection and bleeding. It would also reduce the chance of diagnosing an unimportant cancer that requires treatment. What is the maximum coinsurance that you would pay for an MRI?
a. $100
b. $250

c. $500
d. $1000
e. $2000
f. $3000 or more

7. If you have a positive screening test it is possible to either do more confirmatory blood testing or to do an MRI. More blood testing would allow you to avoid a biopsy at a similar rate compared to an MRI. However an MRI will be more useful in the future as further biopsies can be guided towards areas that are abnormal on the MRI. Getting an MRI would also allow you to see if the lymph nodes are involved and help plan treatment in the event that you are diagnosed with prostate cancer. Assuming the costs are comparable, which would you rather get after a positive screening test?

A. MRI
B. Further blood testing

8. If you needed a biopsy you could either have a biopsy done through the rectum or through the perineum (skin behind scrotum). There is a lower risk of severe infection through the perineum but it is more uncomfortable. Would you rather have the biopsy

a. Rectum (more comfortable)
b. Perineum (lower risk of severe infection)
